# LncRNA PTAR promotes EMT and invasion-metastasis in serous ovarian cancer by competitively binding miR-101-3p to regulate ZEB1 expression

**DOI:** 10.1186/s12943-018-0870-5

**Published:** 2018-08-11

**Authors:** Haihai Liang, Tong Yu, Yue Han, Hua Jiang, Chengyu Wang, Tianyi You, Xiaoguang Zhao, Huitong Shan, Rui Yang, Lida Yang, Hongli Shan, Yunyan Gu

**Affiliations:** 1 Department of Pharmacology (State-Province Key Laboratories of Biomedicine-Pharmaceutics of China, Key Laboratory of Cardiovascular Research, Ministry of Education), College of Pharmacy, Harbin Medical University, Harbin, China; 2 Translational Medicine Research and Cooperation Center of Northern China, Heilongjiang Academy of Medical Sciences, Harbin, 150081 China; 3 Department of Systems Biology, College of Bioinformatics Science and Technology, Harbin Medical University, Harbin, 150001 China; 4 Department of Systems Biology, College of Bioinformatics Science and Technology, Harbin Medical University, Harbin, 150086 China; 5 Training Center for Students Innovation and Entrepreneurship Education, Harbin Medical University, Harbin, 150086 China

**Keywords:** Ovarian cancer, Epithelial-mesenchymal transition, lncRNA PTAR, miR-101, ZEB1

## Abstract

**Background:**

Ovarian cancer (OvCa) is one of the most common malignant diseases of the female reproductive system in the world. The majority of OvCa is diagnosed with metastasis in the abdominal cavity. Epithelial-to-mesenchymal transition (EMT) plays a key role in tumor cell metastasis. However, it is still unclear whether long non-coding RNA (lncRNA) is implicated in EMT and influences cell invasion and metastasis in OvCa.

**Results:**

In this study, using bioinformatcis analysis, we constructed a lncRNA-mediated competing endogenous RNA (ceRNA) network for mesenchymal OvCa and identified lncRNA AP000695.4, which we named pro-transition associated RNA (PTAR). PTAR was significantly up-regulated in the mesenchymal subtype samples compared with the epithelial subtype samples from the TCGA OvCa data sets. In addition, our study showed that PTAR expression was positively correlated with the expression level of ZEB1 in the mesenchymal OvCa samples. Meanwhile, we found that silencing miR-101 promoted cell migration, whereas the overexpression of miR-101 suppressed EMT and cell migration in OvCa cell lines through the regulation of ZEB1. Further analysis showed that enhanced expression of PTAR promoted EMT and metastasis through the regulation of miR-101, whereas silencing PTAR led to the attenuation of TGF-β1-induced tumorigenicity in ovarian cancer cells. Mechanistically, we found that PTAR acted as a ceRNA of miR-101, as forced expression of PTAR reduced the expression and activity of miR-101. More importantly, the knockdown of PTAR reduced tumorigenicity and metastasis in vivo.

**Conclusions:**

Taken together, the results from our study highlight a role for the PTAR-miR-101-ZEB1 axis in OvCa, which offers novel strategies for the prevention of metastasis in OvCa.

## Background

Ovarian cancer (OvCa), one of the most common gynecological malignancies in women worldwide [1], has a high incidence and high mortality rate and is especially difficult to discover during the early stages. This difficulty to discover at the early stages is due to an absence of specific signs or symptoms, as well as a lack of effective or sensitive clinical screening methods for early OvCa [2]. A high rate of recurrence and poor prognosis due to the metastasis of this devastating disease are common and serious problems to be conquered in OvCa [3]. High-grade serous ovarian cancer, the most prevalent type of OvCa, is an aggressive disease with a high rate of relapse and metastasis into the abdominal cavity at later stages [4]. Therefore, more extensive research and a better understanding of the molecular changes that induce the metastasis of tumor cells in serous OvCa is urgently needed.

A growing body of evidence has reported that epithelial-mesenchymal transition (EMT) plays a critical role in cancer cell metastatic dissemination events by endowing cancer cells with a more motile and invasive phenotype [5–7]. Emerging evidence suggests that the acquisition of invasive properties by OvCa cells is accompanied by a loss of epithelial features and a gain of mesenchymal features [7]. In addition to the transforming growth factor-β signaling pathway (TGF-β), which is a major inducer of EMT, the activation of several transcription factors including ZEB1, ZEB2, SNAI1, SNAI2, TWIST1 and TWIST2, either directly or indirectly repress the E-cadherin (CDH1) promoter. Loss of CDH1 expression is considered to be a typical hallmark of EMT [8], however, the molecular events that drive the EMT process during OvCa progression are largely unknown [9].

To date, a large set of non-coding RNAs (ncRNAs) have been recognized as controlling almost every level of gene expression and pathway activation, including the activation and repression of the EMT process [6, 10]. Among them, a surprisingly prominent layer of regulatory macro ncRNAs, long non-coding RNAs (lncRNAs), have been implicated in various types of gene regulation including epigenetic, transcription or post-transcriptional regulation, which contribute to the development of carcinoma and other diseases [11]. Furthermore, accumulating evidence has shown that lncRNAs play a critical role in tumor occurrence, invasion, metastasis and drug resistance by acting as a competitive RNA (ceRNA) for microRNAs (miRNAs) [12, 13]. For example, H19 acts as a molecular sponge for the let-7 family, which are well-characterized tumor suppressors [14–16]. This decrease in let-7 leads to the increased expression of a let-7 target called LIN28, which eventually promotes breast cancer stem cell maintenance [17]. LncRNA ABHD11-AS_1_ promoted ovarian cancer cell proliferation, invasion and metastasis, and inhibited ovarian cancer cell apoptosis by targeting RhoC and its downstream molecules [18]. Li et al. found that lncRNA XIST promoted EMT by regulating ZEB2 via acting as a ceRNA of miR-367/141 in non-small-cell lung cancer [19]**.** However, the role and mechanism of lncRNAs in EMT and their influences on cell invasion and metastasis in OvCa, are still not well understood.

In the present study, we constructed a ceRNA regulatory network for mesenchymal OvCa and identified a lncRNA AP000695.4, which we named pro-transition associated RNA (PTAR), which is up-regulated in mesenchymal OvCa samples. In addition, we found that PTAR down-regulation inhibits OvCa metastasis by up-regulating miR-101, a potential tumor suppressor in OvCa. In conclusion, we hypothesized that PTAR may serve as a competing, endogenous lncRNA by sponging miR-101, which mediates its role in both TGF-β-induced EMT and the invasion-metastasis cascade of OvCa.

## Methods

### Data set

The level 3 gene expression profile and miRNA expression profile for OvCa were downloaded from TCGA (http://gdac.broadinstitute.org/runs/stddata__2016_01_28/data/OV/20160128//), which had integrated epithelial (iE) and integrated mesenchymal (iM) subtype labels [20]. The processed, sequenced, expression profiles of lncRNAs in the TCGA OvCa dataset were obtained in The Atlas of Non-coding RNAs in Cancer (TANRIC) database [21]. The miRNA-mRNA interactions and miRNA-lncRNA interactions were collected from the database at http://www.medsysbio.org/EMTRegulome, which systematically collects the regulations among transcription factors, miRNA, lncRNA and EMT genes and records diverse regulatory motifs involved in the EMT process.

### Identification of ceRNAs involved in mesenchymal OvCa

T tests were performed to detect differentially expressed (DE) genes, lncRNAs and miRNAs in the iM subtype compared with the iE subtype of OvCa. The *P* values were corrected using the Benjamini-Hochberg method. The Pearson’s correlation test was used to calculate the correlation between the expression of DE miRNAs and DE lncRNAs or DE genes. A hypergeometric distribution model was used to test whether the DE EMT genes shared a significant number of miRNA binding sites with DE lncRNAs. The mesenchymal-related ceRNAs were selected according to the following criteria: (1) The EMT coding-genes, lncRNAs and miRNAs were significantly, differentially expressed in iM OvCa samples compared with iE OvCa samples under the constraint of a false discovery rate (FDR) < 0.1. (2) The expression of DE EMT coding-genes (lncRNAs) and DE miRNAs were significantly correlated (*P* < 0.05, Pearson’s correlation test) in iM OvCa samples, while not correlated in iE OvCa samples. The ceRNA network was presented using the Cytoscape web tool (http://js.cytoscape.org). All analyses were performed in R 3.2.3 (https://www.r-project.org/).

### Cell culture and treatment

The human ovarian cancer cell lines SKOV3, A2780 and OVCAR3 were obtained from the Cell Bank of the Chinese Academy of Sciences (Shanghai, China). The SKOV3 and OVCAR3 cells were cultured in RPMI-1640 medium (Biological Industries, Kibbutz Beit-Haemek, Israel), and A2780 cells were cultured with DMEM (Biological Industries, Kibbutz Beit-Haemek, Israel). All the cells were supplemented with 10% fetal bovine serum (FBS, Biological Industries, Kibbutz Beit-Haemek, Israel), 1% penicillin/streptomycin (Beyotime, Jiangsu, China) and incubated at 37 °C with 5% CO_2._

### Plasmid construct and transfection

For PTAR overexpression, the full-length PTAR cDNA was amplified and subcloned into pcDNA3.1. An empty vector was used as a negative control. Three shRNAs targeting PTAR were synthesized for PTAR knockdown, and a scrambled shRNA was synthesized for the negative control. All plasmids were isolated using AxyPrep DNA Miniprep Kit (Axygen, Scientific, Union City, CA, USA). A hsa-miR-101 mimic was used in place of miR-101, a chemically modified antisense oligonucleotide (antagomir AMO-101) was used to inhibit miR-101 expression, and a scrambled oligonucleotide (GenePharma) was used as a control. The hsa-miR-101 mimic, inhibitor and stable negative control were purchased from GenePharm (Shanghai, China). For transfection, cells were cultured in six-well plates until 70% confluence. The plasmids were then transfected using Lipofectamine 2000 (Thermo Fisher Scientific, Waltham, MA, USA) based on the manufacturer’s instructions. Six hours after transfection, hsa-miR-101 mimics/inhibitors were added to the cells and incubated for 48 h.

### Wound healing assay

The cells were cultured in six-well plates (Nest, Biotechnology, Jiangsu, China) overnight. The cells monolayers were wounded by scratching with plastic 10-μl micropipette tips and washed 2 times with PBS. Fresh growth medium with no FBS was added to the plates. Subsequently, the cells were transfected as described above for 48 h. Images of the different stages of wound healing were photographed via microscopy at 0, 24 and 48 h. Relative cell motility was quantified using Image-Pro Plus.

### Transwell migration and invasion

Cells were seeded in the upper chamber of transwell plates (Corning, NY, USA) with serum-free medium. Ten percent FBS medium was added to the lower chamber of the transwell. Next, cells were transfected as described above for 48 h. For the invasion experiments, the upper chamber was covered with RPIM-1640 and matrigel (BD Biosciences, San Jose, CA) mixture. Finally, cells on the top of the chamber were removed with cotton swabs, while cells that went through the membrane were stained with 0.5% crystal violet (Sigma-Aldrich, St. Louis, MO) observed and counted under a microscope at 100 x magnification.

### Real-time quantitative reverse transcription PCR (qRT-PCR)

Total RNA was extracted from tumor tissues that were collected from nude mice and SKOV3/A2780 cells using a Trizol reagent (Thermo Fisher Scientific). The cDNA was synthesized using a cDNA Reverse Transcription Kit (Applied Biological Materials Inc., Canada). The expression levels of mRNAs and miRNAs were quantified using a 7500 Fast Real-Time PCR System with a SYBR green PCR Master Mix. The relative mRNA levels were determined using the comparative Ct method with GAPDH or U6 as the reference gene, and the formula 2^-ΔΔCt.^

### Western blot analysis

For western blot analysis, total protein samples were extracted from SKOV3, A2780 or OVCAR3 cells and tumor tissues collected from nude mice using RIPA lysis buffer with a protease inhibitor (Beyotime, Jiangsu, China). An equal amount of protein (60 μg/lane) was loaded on 8% SDS-polyacrylamide gels and then transferred to a Pure Nitrocellulose Blotting membrane (Pall Life Science, AZ, USA). Next, the membrane was blocked in 5% non-fat milk for 1.5 h and then probed with primary antibodies against ZEB1 (1:1000, Abcam, Cambridge, USA), E-cadherin (1:500, Proteintech, Rosemont, IL, USA), vimentin (1:1000, Cell Signaling Technology, Beverly, MA), fibronectin 1 (FN1, 1:500, Proteintech, Rosemont, IL, USA), and GAPDH (1:2000, Kangchen, Shanghai, China) overnight at 4 °C. Then, the membranes were incubated with anti-mouse or anti-rabbit secondary antibodies (1:8000; LI-COR Biosciences). Finally, immunoreactivity was detected using the Odyssey Infrared Imaging System (Gene Company Limited, Hong Kong, China).

### Lentiviral vector construct and tumor xenografts in mice

The lentiviral constructs of PTAR shRNA were constructed by Biowit Technology (Shenzhen, China). The nude mice were infected with the constructed lentiviral vectors at day 7 following tumor xenografts. 10^6^ SKOV3 cells were injected into 4-week old, female, athymic BALB/c nude mice which were obtained from SLRC Laboratory Animals (Shanghai, China), intraperitoneally. The nude mice were housed under specific pathogen-free conditions and sacrificed after 28 days. Then, the xenograft tumor tissues were harvested, weighed, formalin-fixed and paraffin-embedded for subsequent immunohistochemical staining. The remaining tumor tissues were stored in a − 80 °C refrigerator for analysis of protein and RNA levels. The procedures were in accordance with the regulations of the Ethics Committee of Harbin Medical University and the Guide for the Care and Use of Laboratory Animals published by the US National Institutes of Health (NIH Publication No. 85–23, 2011).

### Immunohistochemistry (IHC) analysis

Cancer tissue sections were de-paraffinized in a xylene series and rehydrated through a series of descending ethanol concentrations. After washing with double-distilled H_2_O, the sections were treated with 3% H_2_O_2_ in methanol for 30 min and blocked with normal goat serum for 1 h. Then, the sections were incubated with anti-E-cadherin (Proteintech, Wuhan, China), anti-FN1 (Proteintech, Wuhan, China), anti-ZEB1 (Abcam, USA), and anti-vimentin (Cell Signaling Technology, MA) overnight at 4 °C and then washed in PBS and incubated with secondary antibodies for 30 min at room temperature. Next, the sections were stained with a DAB staining solution and counterstained with hematoxylin for 2 min. Images were taken under an Olympus IX73 microscope (Olympus, Valley, PA).

### Statistical analysis

All data were expressed as the mean ± SEM. Statistical analyses were performed using Student’s t-test for two group comparisons. One-way analysis of variance ANOVA followed by Dunnett’s post hoc test were used for multiple comparisons. GraphPad Prism 5.0 was used for statistical analyses. A value *P* < 0.05 was considered statistically significant.

## Results

### Constructions of a ceRNA network for mesenchymal ovarian cancer

**Fig. 1:**
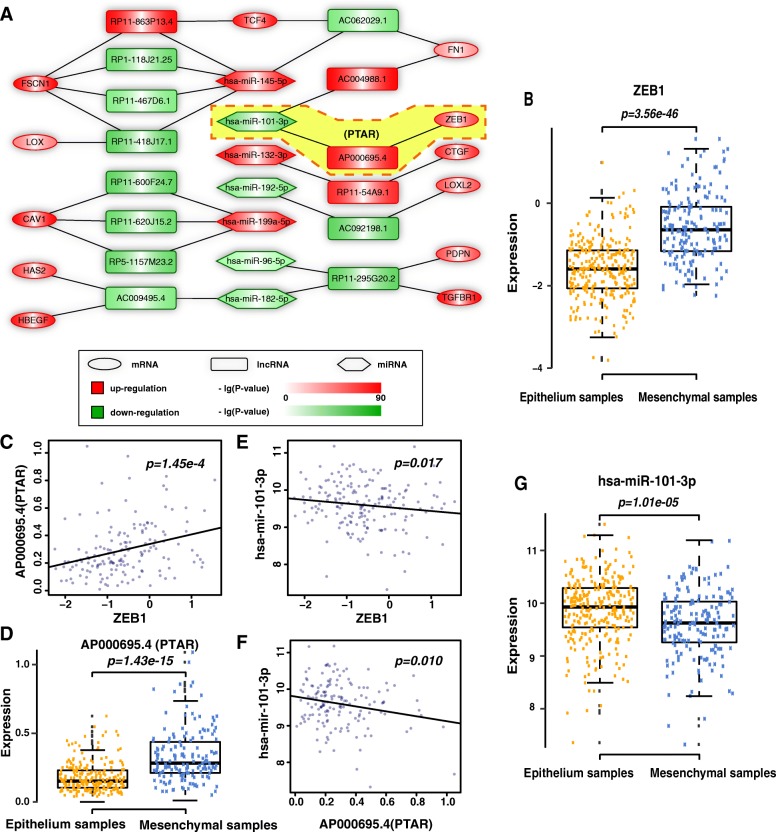
The identification of a ceRNA network for mesenchymal OvCa. **a** The ceRNA network in mesenchymal OvCa. Rounded rectangles, ellipses and hexagons denote lncRNAs, EMT genes and miRNAs, respectively. Up-regulated lncRNAs, EMT genes and miRNAs are shown in red and down-regulated lncRNAs, EMT genes and miRNAs are shown in green. **b** ZEB1 was significantly up-regulated in mesenchymal OvCa compared with epithelial OvCa in the TCGA data set. **c** Positive correlation between PTAR and ZEB1 expression in the TCGA data set. **d** The lncRNA PTAR was significantly up-regulated in mesenchymal OvCa compared with epithelial OvCa in the TCGA data set. **e** Nagative correlation between miR-101 and ZEB1 expression in the TCGA data set. **f** Nagetive correlation between PTAR and miR-101 expression in the TCGA data set. **g** miR-101 was significantly down-regulated in mesenchymal OvCa compared with epithelial OvCa in the TCGA data set

The iM subtype specific ceRNA network for OvCa contains 7 miRNAs, 14 lncRNAs and 12 EMT-related coding-genes (Fig. 1a). A total of 19 lncRNA-EMT gene pairs had a significantly higher number of common miRNAs than would be expected by chance (*P* < 0.05, hypergeometric test). According to the assumption of ceRNA, the expression of the EMT gene and miRNA, and lncRNA and miRNA should show a negative correlation. The expression of lncRNA and the EMT-gene should show a positive correlation. Using a correlation *P*-value < 0.05, we identified two ceRNA pairs which are ZEB1, miR-101-3p and lncRNA AP000695.4, fibronectin 1 (FN1), and miR-101-3p, lncRNA AC004988.1. The gene ZEB1 encodes a transcription factor that represses E-cadherin transcription [12]. E-cadherin is a critical protein that is associated with an epithelial cell phenotype. The down-regulation of E-cadherin is believed to be a driving event for EMT, which is involved in cancer invasion and metastasis [22]. We found that ZEB1 was significantly up-regulated in the iM OvCa samples (*P* = 3.56 × 10^− 46^, t test, Fig. 1b). In the mesenchymal-related ceRNA network, ZEB1 expression was positively correlated with the expression level of lncRNA AP000695.4 (ENSG00000233818.1) in the iM OvCa samples (*P* = 1.45 × 10^− 4^, Pearson’s correlation test, Fig. 1c). AP000695.4 was significantly up-regulated in the iM subtype samples compared with the iE subtype samples in the TCGA data sets (*P* = 1.43 × 10^− 15^, t test, Fig. 1d). The 3’-UTRs of ZEB1 and lncRNA AP000695.4 significantly had common binding sites with miR-101-3p. Moreover, both ZEB1 and lncRNA AP000695.4 were negatively correlated with the expression level of miR-101-3p in the iM OvCa samples (*P* = 0.017 for ZEB1 in Fig. 1e and *P* = 0.010 for lncRNA AP000695.4 in Fig. 1f, Pearson’s correlation test). Yang et al. reported that miR-101 is one of the key miRNAs that regulates the largest number of iM-related genes [20]. Moreover, miR-101-3p was significantly down-regulated in the iM subtype (*P* = 1.01 × 10^− 5^, t test, Fig. 1g). Thus, we hypothesized that AP000695.4 up-regulates ZEB1 by competitively binding to miR-101 resulting in EMT induction and OvCa cell invasion. For convenience, we refer to AP000695.4 as the pro-transition associated RNA (PTAR) in this study.

### LncRNA PTAR knockdown reduces tumorigenicity and metastasis in vivo

**Fig. 2:**
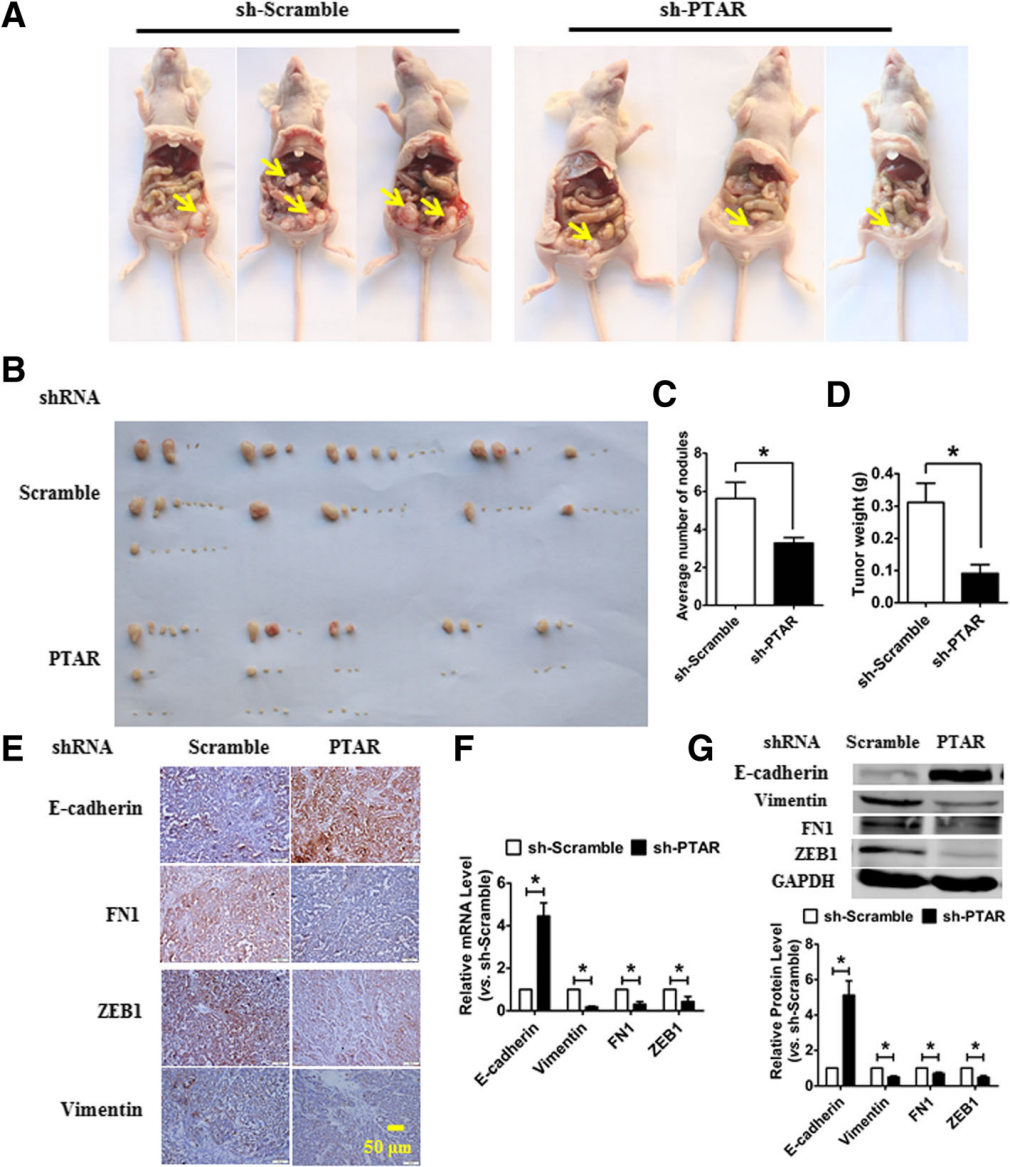
Knockdown of lncRNA PTAR inhibits tumor progression in an orthotopic mouse model of OvCa. **a** The macroscopic observation of the size and range of metastatic lesions in nude mice. **b** Representative images of tumors from nude mice bearing xenograft tumors with SKOV3 cells infected with a lentivirus carrying a short hairpin RNA against PTAR (lenti-sh-PTAR) or its scramble negative control (lenti-sh-Scramble). **c**, **d** The average number of metastatic nodules and tumor xenograft weights in nude mice. *n* = 11–13. *P < 0.05. **e** An immunohistochemistry assay was applied to determine the expression of E-cadherin, fibronectin 1 (FN1), zinc finger E-box binding homeobox 1 (ZEB1) and vimentin in a cell-derived xenograft tumor model. **f**, **g** The expression of epithelial-mesenchymal transition (EMT) relevant markers, including E-cadherin, vimentin, FN1 and ZEB1 in tissues derived from xenograft tumors, as determined by qRT-PCR and western blot, respectively. *n* = 6. *P < 0.05

To examine the effect of PTAR on ovarian cancer tumorigenicity and metastasis, a nude mouse xenograft tumor model was constructed. Macroscopic observation of the intraperitoneally injected nude mice revealed that the size and total number of tumor nodes were higher in the sh-PTAR infected mice than in the sh-Scramble nude mice (Fig. 2a and b). Meanwhile, the average number of harvested nodules (Fig. 2c) and tumor weight (Fig. 2d) were also significantly decreased in PTAR knockdown mice compared with the sh-Scramble group. Furthermore, the tumor sections were stained for E-cadherin, FN1, ZEB1 and vimentin expression to quantitatively evaluate the EMT associated marker. Here, we observed that tumors derived from sh-PTAR group displayed increased E-cadherin expression and reduced FN1, ZEB1 and vimentin expression (Fig. 2e). Meanwhile, qRT-PCR and western blot assays showed that the mRNA and protein levels of the epithelial marker E-cadherin were markedly increased and accompanied by a decrease in the mesenchymal marker vimentin, FN1 and ZEB1 in xenograft tumors from the sh-PTAR mice (Fig. 2f and g).

### Overexpression of PTAR promotes EMT and metastasis through the regulation of miR-101

**Fig. 3:**
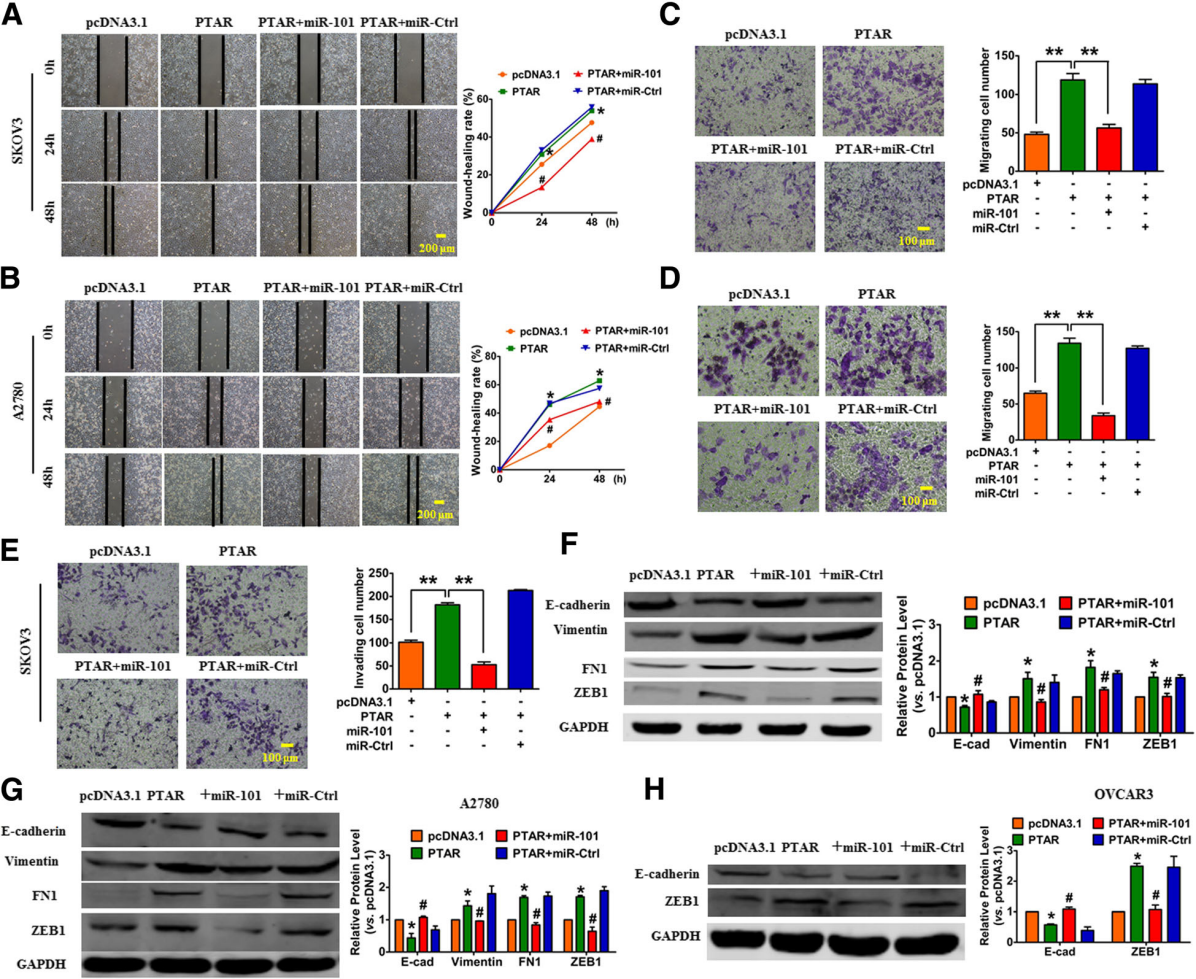
PTAR promotes EMT and metastasis through the modulation of miR-101/ZEB1. The SKOV3, A2780 and OVCAR3 cells were transfected with PTAR plasmid with or without miR-101 mimic. a, **b** Wound healing assays showing cell migration in SKOV3 and A2780 cells. *n* = 4, *P < 0.05 vs. pcDNA3.1, ^#^*P* < 0.05 vs. PTAR. **c**, **d** Migration assays in SKOV3 and A2780 cells, respectively. **e** The invasiveness of SKOV3 cells was determined by an invasion assay using matrigel transwell chambers. *n* = 4, **p < 0.01. **f** The protein levels of E-cadherin, vimentin, FN1 and ZEB1 in SKOV3 cells. *n* = 5, *P < 0.05 vs. pcDNA3.1, ^#^*P* < 0.05 vs. PTAR. **g** The protein levels of E-cadherin, vimentin, FN1 and ZEB1 in A2780 cells. *n* = 5, *P < 0.05 vs. pcDNA3.1, ^#^*P* < 0.05 vs. PTAR. **h** The protein levels of E-cadherin and ZEB1 in OVCAR3 cells. *n* = 3, *P < 0.05 vs. pcDNA3.1, ^#^*P* < 0.05 vs. PTAR

Next, we transfected the PTAR overexpression vector into the OvCa cells to determine whether miR-101 mediates the effects of PTAR on EMT, cell migration and invasion. The wound healing assay showed that the overexpression of PTAR enhanced the cell migratory capacity both in SKOV3 and A2780 cells, whereas cell migration was blocked by miR-101 treatment (Fig. 3a and b). In addition, the transwell assay revealed a clear decrease in migratory capacity of cells that were transfected with PTAR and miR-101 compared with PTAR alone (Fig. 3c and d). Moreover, the invasion assay in SKOV3 cells revealed PTAR markedly promoted cell invasion when compared with the empty vector pcDNA3.1 transfection. This effect was abolished by miR-101 co-transfection (Fig. 3e). Consistent with these results, we found that the overexpression of PTAR led to a decreased expression of E-cadherin but an increased expression of vimentin, FN1 and ZEB1 in SKOV3 and A2780 cell lines, indicating that the epithelial cells acquired mesenchymal properties. However, these mesenchymal properties were reversed by miR-101 mimics (Fig. 3f and g). In addition, we also found that forced expression of PTAR resulted in the down-regulation of E-cadherin and up-regulation of ZEB1 in OVCAR3 cells, and which were reversed after overexpression of miR-101 (Fig. 3h).

**Fig. 4:**
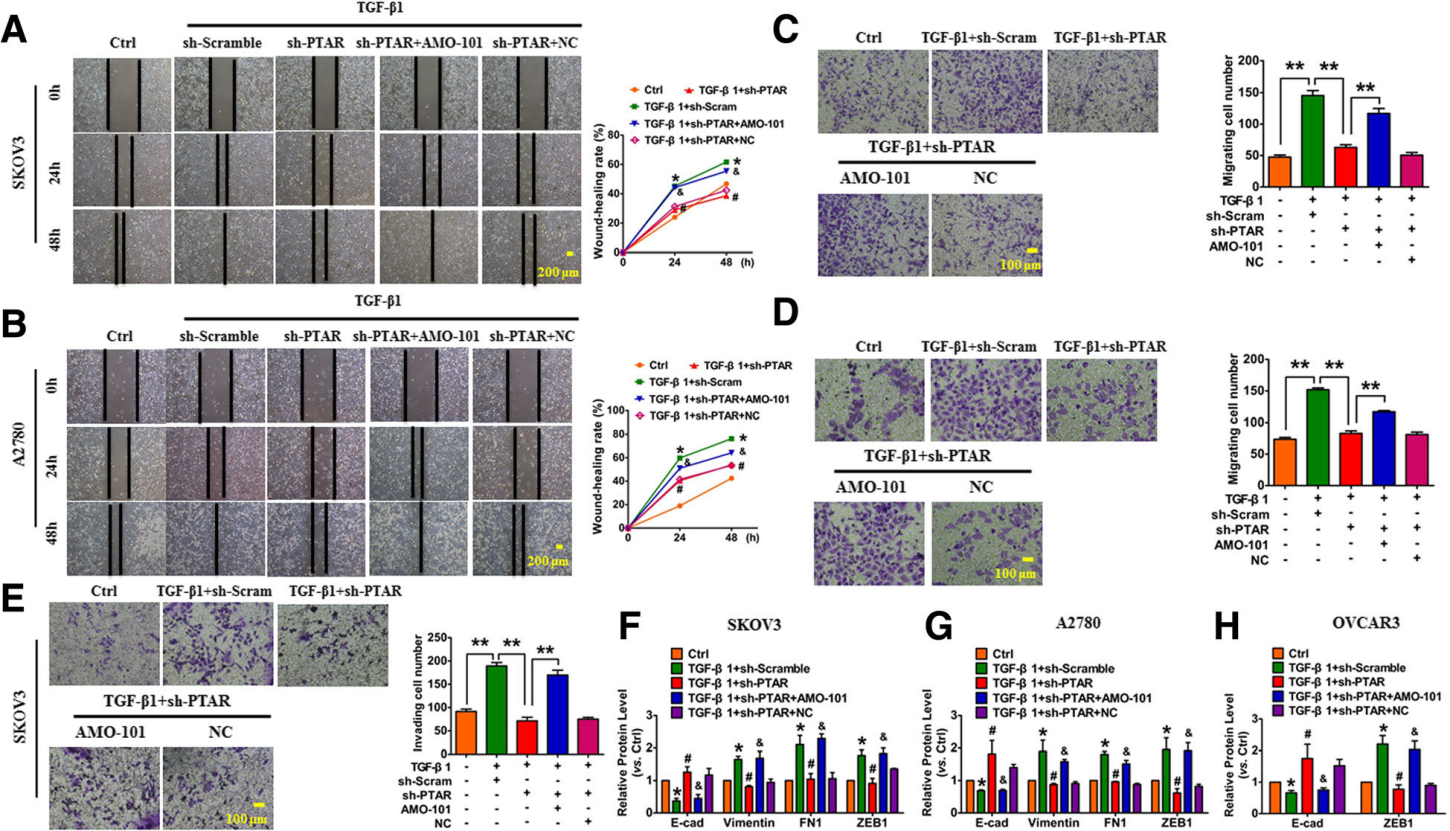
Silencing PTAR alleviates ovarian cancer cell tumorigenicity. The SKOV3, A2780 and OVCAR3 cells were transfected with sh-PTAR with or without miR-101 inhibition in the presence or absence of 10 ng/ml TGF-β1 for 48 h. **a**, **b** Wound healing assays were performed both in SKOV3 and A2780 cells treated as specified. *n* = 4, *P < 0.05 vs. Ctrl, ^#^*P* < 0.05 vs. TGF-β1 + sh-Scram, ^&^*P* < 0.05 vs. TGF-β1 + sh-PTAR. **c**, **d** Cell migration in SKOV3 and A2780 cells was determined using transwell chambers. *n* = 4, ** *P* < 0.01. **e** The invasion ability of SKOV3 cells was evaluated using matrigel transwell chambers. *n* = 4, ** *P* < 0.01. **f**, **g** The protein levels of E-cadherin, vimentin, FN1 and ZEB1 in SKOV3 and A2780 cells. *n* = 5, *P < 0.05 vs. Ctrl, ^#^*P* < 0.05 vs. TGF-β1 + sh-Scram, ^&^*P* < 0.05 vs. TGF-β1 + sh-PTAR. **h** The protein levels of E-cadherin and ZEB1 in OVCAR3 cells. *n* = 3, *P < 0.05 vs. Ctrl, ^#^*P* < 0.05 vs. TGF-β1 + sh-Scram, ^&^*P* < 0.05 vs. TGF-β1 + sh-PTAR

To further analyze the effect of PTAR on tumorigenicity in ovarian carcinoma, as well as determine if this effect was mediated by miR-101, the SKOV3 and A2780 cells were transfected with miR-101 inhibitor and inhibitor negative control (NC), respectively. Wound healing and transwell assays demonstrated that the knockdown of PTAR attenuated TGF-β1-promoted migration in both the SKOV3 and A2780 cells, which was reversed by AMO-101 treatment (Fig. 4a-d). Furthermore, the invasion assay indicated that the knockdown of PTAR restrained TGF-β1-induced cell invasion, but the co-transfection of sh-PTAR and AMO-101 restored the ability of cells to invade (Fig. 4e). In addition, western blot assays demonstrated that knockdown of PTAR inhibited TGF-β1-induced up-regulation of several mesenchymal markers, and increased TGF-β1-induced down-regulation of epithelial marker E-cadherin in SKOV3, A2780 and OVCAR3 cell lines. Importantly, the effects of PTAR silencing were effectively reversed by AMO-101 co-transfection (Fig. 4f-h). These data suggest that the silencing of PTAR attenuates tumorigenicity in ovarian cancer cells.

### PTAR acts as a ceRNA to negatively regulate miR-101 expression and activity

**Fig. 5:**
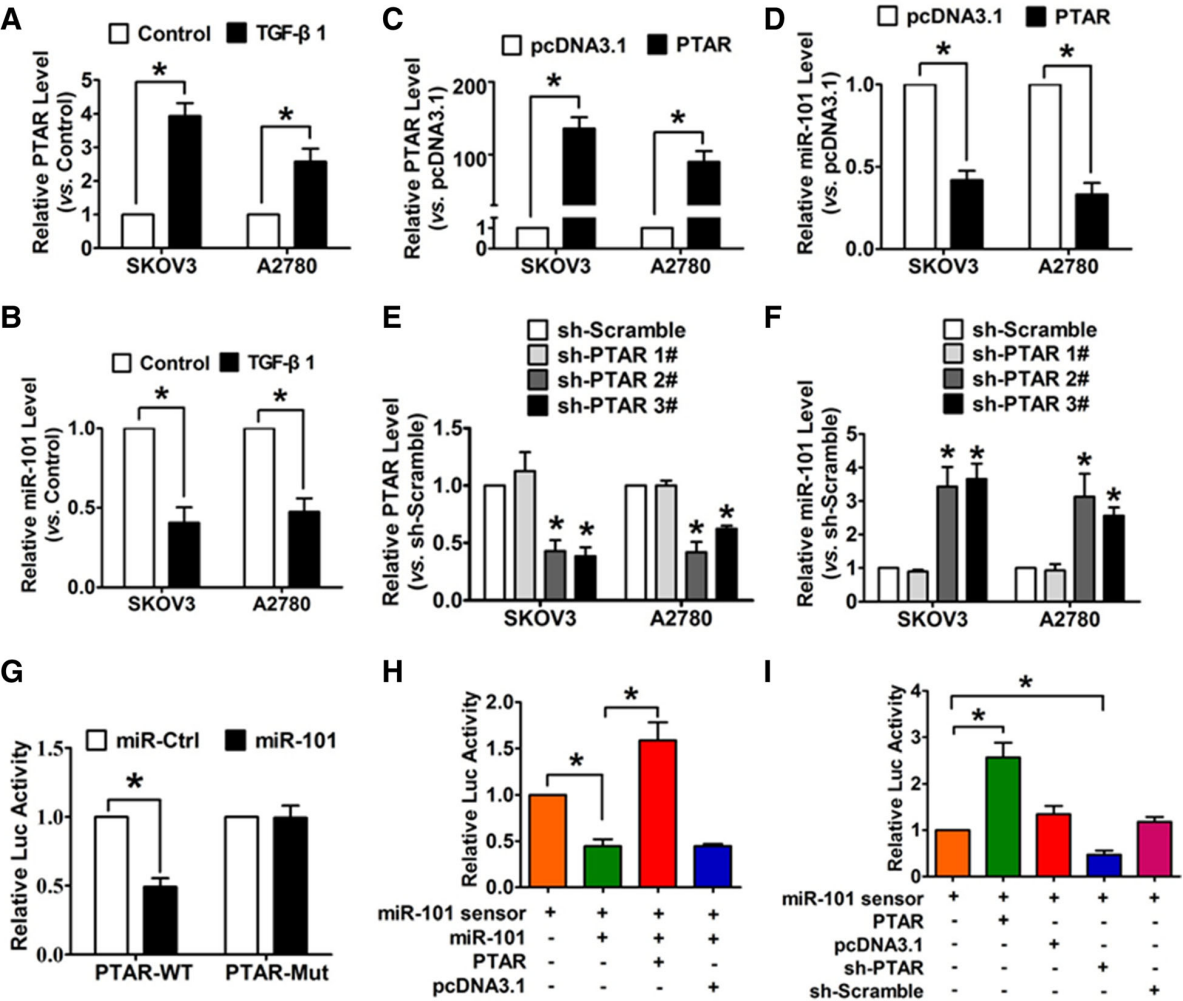
PTAR negatively regulates miR-101 expression and activity in OvCa cells. **a**, **b** The expression of PTAR and miR-101 in SKOV3 and A2780 cells after treatment with 10 ng/ml TGF-β1 for 48 h. **c**, **d** Analysis with qRT-PCR was used to assess the expression of PTAR and miR-101 in SKOV3 and A2780 cells in response to PTAR overexpression. **e** The knockdown efficiency of the sh-PTAR plasmid was confirmed by qRT-PCR analysis. **f** Expression of miR-101 was detected in OvCa cells in response to PTAR suppression. **g** The luciferase reporter activity of chimeric vectors carrying the luciferase gene and a fragment of PTAR containing the (WT) binding site or a mutated binding site for miR-101. **h**, **i** The luciferase activity of miR-101 sensor in OvCa cells transfected as specified. *n* = 5. *P < 0.05

Next, we examined whether PTAR regulates the expression and activity of miR-101 through acting as a ceRNA of miR-101. As shown in Fig. 5a and b, we found that the expression of PTAR was significantly up-regulated in both SKOV3 and A2780 cells in response to 10 ng/ml of TGF-β1 for 48 h. This was also accompanied by a reduction of miR-101 levels. In addition, the PTAR overexpression plasmid was successfully constructed (Fig. 5c) and showed that ectopic expression of PTAR markedly reduced miR-101 levels in both SKOV3 and A2780 cells **(**Fig. 5d). In contrast, we designed shRNAs that targeted and knockdown PTAR. This was validated in sh-PTAR 2# and 3# (Fig. 5e). The qRT-PCR results showed that the knockdown of PTAR with sh-PTAR 2# and 3# enhanced the expression of miR-101, respectively (Fig. 5f). Additionally, we constructed luciferase reporter vectors that contained wild-type or mutant miR-101 putative binding sites in PTAR. As shown in Fig. 5g, the relative luciferase activity in cells co-transfected with PTAR-WT and miR-101 was approximately 50% of the cells with PTAR-WT and miR-Ctrl co-transfection. This luciferase activity was abolished when the potential miR-101 binding site was mutated. Moreover, the miR-101 sensor reporter vector was constructed and showed an increase of luciferase activity in SKOV3 cells with PTAR transfection. This indicates that PTAR binds to miR-101 to limit the inhibitory effects of miR-101 on luciferase activity (Fig. 5h). In contrast, the silencing of PTAR by shRNA inhibited the luciferase activity of the miR-101 sensor. Furthermore, the overexpression of PTAR alleviated the inhibitory effects of miR-101 on its sensor (Fig. 5i). These results indicate that PTAR inhibits the expression and activity of miR-101 by acting as a ceRNA.

### EMT and cell migration are suppressed by miR-101 in OvCa cell lines by directly targeting ZEB1

**Fig. 6:**
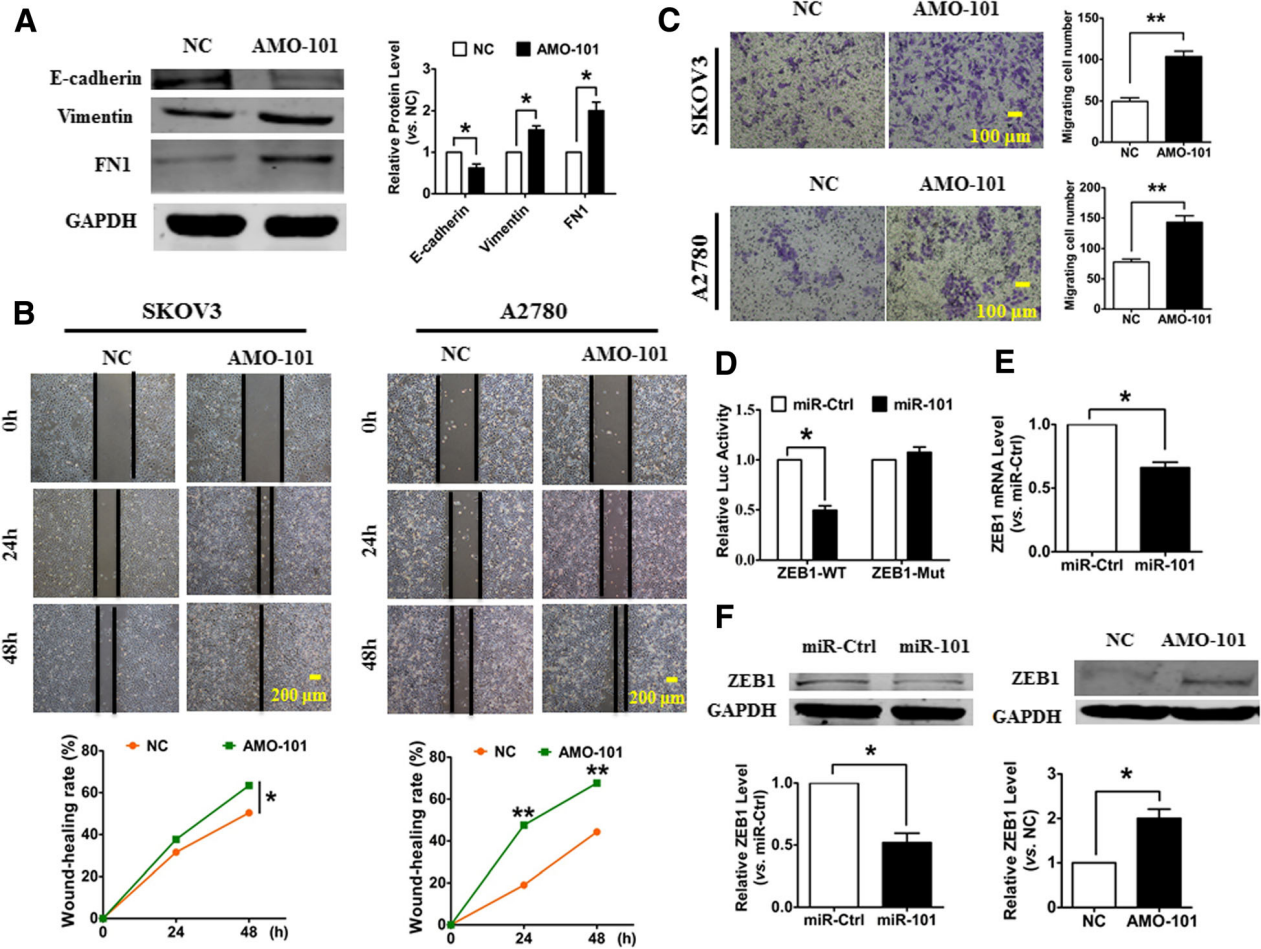
Silencing miR-101 promotes OvCa cell migration by regulating ZEB1. **a** The western blot analysis of epithelial and mesenchymal markers in SKOV3 cells transfected with AMO-101. Wound-healing **b** and migration assays **c** were applied to determine the effect of miR-101 knockdown on OvCa cell migration. **d** The luciferase reporter activity of chimeric vectors carrying the luciferase gene and a fragment of the ZEB1 3′-UTR containing the wild-type or mutated miR-101 binding site. **e** The overexpression of miR-101 inhibited the mRNA expression of ZEB1 in SKOV3 cells. **f** The overexpression of miR-101 repressed the protein expression of ZEB1 in SKOV3 cells, whereas knockdown of miR-101 had the opposite effect. *n* = 5. *P < 0.05, **P < 0.01

EMT was reported to be suppressed by miR-101 in hepatocellular carcinoma and ovarian carcinoma [13, 23]. Here, to examine whether miR-101 inhibition promotes EMT in OvCa cells, we transfected AMO-101 into OvCa cells in order to silence miR-101 expression. As shown in Fig. 6a**,** the knockdown of miR-101 in SKOV3 cells resulted in the dysregulation of several EMT-associated markers, including decreased E-cadherin and increased vimentin and FN1. Moreover, a wound-healing assay showed that miR-101 inhibition promoted the migration of both SKOV3 cells and A2780 cells (Fig. 6b). Consistent with these results, a migration assay revealed that the silencing of miR-101 induced OvCa cell migration (Fig. 6c). To investigate if ZEB1 mediated the pro-migration effect of miR-101 silencing, we constructed pGL3 luciferase expression vectors carrying a fragment of the ZEB1 3′UTR that contains either the wild type (WT) or mutated miR-101 binding sites. As depicted in Fig. 6d, the enhanced expression of miR-101 inhibited the luciferase activity observed after transfection with WT pGL3-ZEB1; however, it failed to suppress the luciferase activity when the putative miR-101 binding site was mutated (pGL3-ZEB1-Mut). Further studies showed that the overexpression of miR-101 inhibited the expression of ZEB1 at the mRNA level in SKOV3 cells (Fig. 6e). Meanwhile, the forced expression of miR-101 inhibited the protein level of ZEB1 in SKOV3 cells, whereas the silencing of miR-101 had the opposite effect (Fig. 6f). These results suggest that ZEB1 is one of the direct targets of miR-101 and mediates the effects of miR-101 on EMT and migration in OvCa cells.

**Fig. 7:**
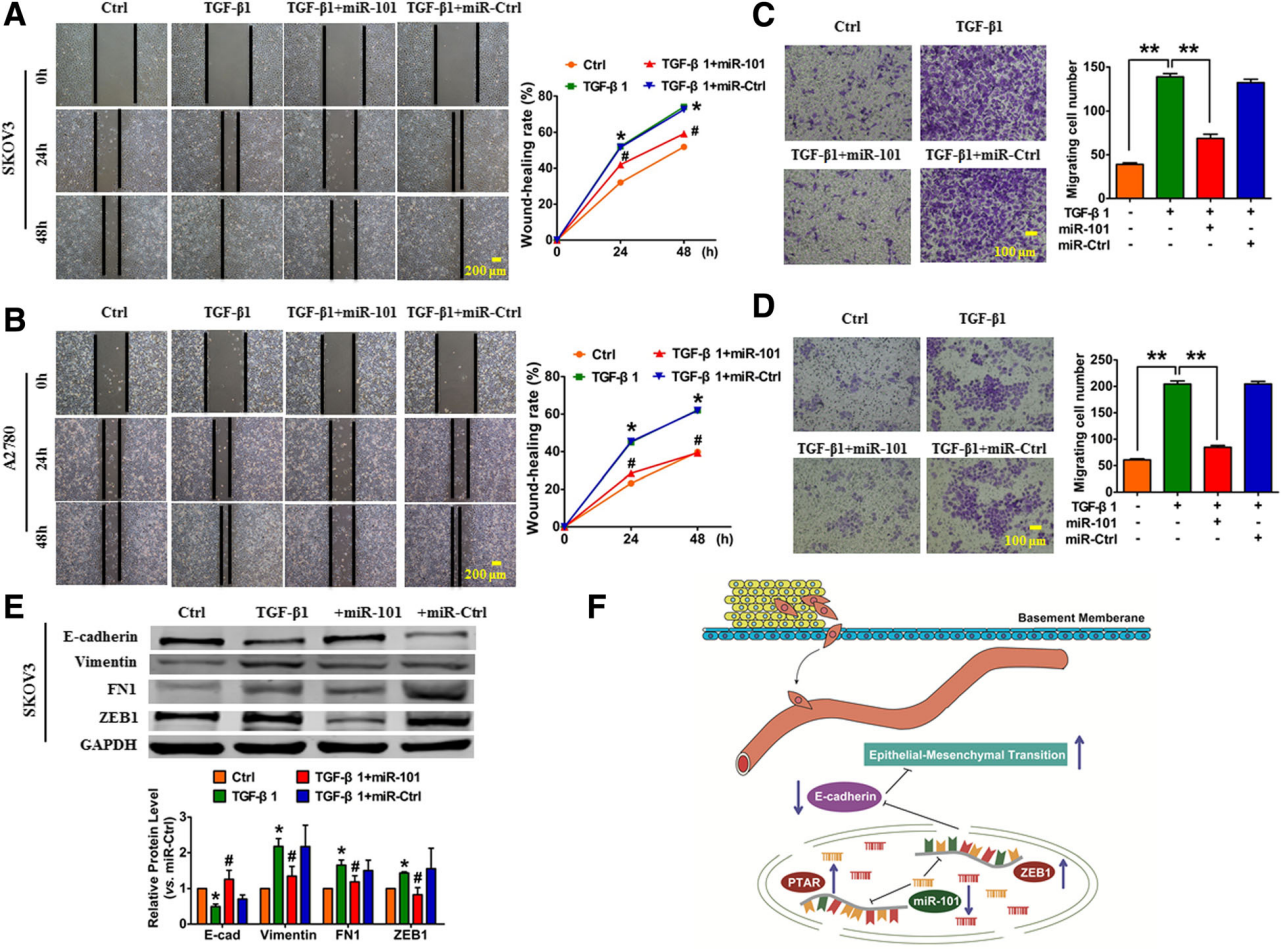
Overexpression of miR-101 attenuated TGF-β1-induced metastasis in OvCa cell lines. **a**, **b** Wound healing assays performed in SKOV3 and A2780 cells, respectively. Images were captured at 0, 24, and 48 h. *n* = 4, *P < 0.05 vs. Ctrl, ^#^*P* < 0.05 vs. TGF-β1. **c**, **d** The migration ability of SKOV3 and A2780 cells was determined using transwell assays. *n* = 4, **P < 0.01. **e** The expression levels of epithelial and mesenchymal markers were detected by western blot in SKOV3 cells. *n* = 5, *P < 0.05 vs. Ctrl, ^#^*P* < 0.05 vs. TGF-β1. **f** Up-regulation of lncRNA PTAR enhanced ZEB1 expression by competitively binding miR-101, which promoted OvCa cell EMT and invasion

To further verify the function of miR-101, we transfected SKOV3 and A2780 cells with either miR-101 mimic or miR-Ctrl. First, a wound healing assay was performed to evaluate the effect of miR-101 on cell migration in OvCa cells. As shown in Fig. 7a and b, the ectopic expression of miR-101 reduced the cell migratory capacity both in the SKOV3 and A2780 cells. Consistently, transwell assays confirmed that miR-101 overexpression inhibited TGF-β1-induced cell migration compared with miR-Ctrl transfected cells (Fig. 7c and d). Furthermore, the data from western blots showed that the forced expression of miR-101 restored the TGF-β1-induced down-regulation of E-cadherin protein levels, whereas it reduced the up-regulation of the mesenchymal markers vimentin, FN1 and ZEB1 in SKOV3 cells (Fig. 7e).

## Discussion

In the current study, we showed that the lncRNA PTAR is a key regulator of EMT and promotes the OvCa invasion-metastasis cascade. We determined this using evidence from systems-based miRNA, lncRNA and mRNA analyses of large-scale OvCa data sets, along with in vivo (nude mice) and in vitro (cell lines) experiments. The up-regulation of PTAR led to an elevated expression of ZEB1, which is a transcriptional repressor of E-cadherin, by competitively binding to miR-101. This binding of PTAR to miR-101 resulted in the promotion of OvCa cell EMT and invasion. We demonstrated that miR-101 inhibits the mesenchymal phenotype, as well as the TGF-β1-induced EMT, at least in part, via directly targeting ZEB1. We established the importance of PTAR-miR-101-ZEB1 in the EMT process by showing a positive correlation between PTAR and ZEB1 expression and inverse correlations between miR-101 and PTAR expression and miR-101 and ZEB1 expression in iM OvCa samples (Fig. 7f). Our findings not only suggest an important role of lncRNAs acting as miRNA sponges during the regulation of EMT progression in OvCa but also highlight the potential of lncRNA PTAR to act as a target for EMT inhibition in OvCa.

To date, a variety of miRNAs have been reported to be involved in the pathogenesis of ovarian carcinoma [24–26]. Yang et al. reported that miR-506 targeted the regulation of SNA12, which led to the inhibition of tumor growth by promoting E-cadherin expression [20]. In addition, colleagues from their labs also found that high expression of miR-506 was associated with longer progression-free periods and overall survival. miR-506 could also be a potential marker for patients’ response to chemotherapy in serous ovarian cancers [27]. In a previous study, miR-101 had been shown to suppress EMT by targeting ZEB1 and ZEB2 in ovarian carcinoma [23]. Consistently, we also confirmed a tumor suppressing function of miR-101, as indicated by reduced EMT progression, cell invasion and metastatic ability. However, the up-stream regulators for the dysregulation of these miRNAs in OvCa are not well understood.

Increasingly more studies have reported that interactions between lncRNAs and miRNAs play a critical role in cancer progression and metastasis. For instance, in colorectal cancer, lncRNA UICLM acts as a ceRNA for miR-215 to regulate ZEB2 expression and promote colorectal cancer metastasis [28]. lncRNA-PAGBC promotes tumor growth and metastasis of gallbladder cancer cells by competitively binding to the tumor suppressive microRNAs, miR-133b and miR-511 [29]. Long non-coding RNA linc00673 regulated non-small cell lung cancer proliferation, migration, invasion and epithelial mesenchymal transition by inhibiting miR-150-5p and modulate ZEB1 expression [30]. In a recent study, MALAT1 was found to compete with endogenous RNA to regulate ZEB2 expression by sponging with miR-200 s in kidney carcinoma [31]. In the current study, we tried to examine the role and mechanism of lncRNAs in OvCa metastasis by constructing a ceRNA network for mesenchymal OvCa. Here, through bioinformatics analysis, we identified an inverse relationship between miR-101 and PTAR expression levels in ovarian cancer tissues and cell lines. Additionally, dual-luciferase reporter assays confirmed the interaction between miR-101 and PTAR. Therefore, we hypothesized that miR-101 is at least partially required for PTAR’s oncogenic effects. As expected, functional studies verified that PTAR promoted EMT and migration of OvCa cells, which was blocked by miR-101 application. Moreover, PTAR knockdown attenuated TGF-β1-induced EMT and metastasis, while an miR-101 inhibitor reversed this effect. Surprisingly, a positive relationship between PTAR and ZEB1 was observed in high-throughput data sets, and it was concluded that PTAR could regulate ZEB1 expression via the modulation of miR-101.

Notably, we also revealed another key ceRNA relationship, which is the lncRNA AC004988.1 and FN1. Both AC004988.1 and FN1 have binding sites with miR-101 and showed a negative correlation of expression levels in TCGA data sets. These results indicate that miR-101 regulates the EMT process not only by targeting ZEB1, but also FN1. Other lncRNAs, such as lncRNA AC004988.1, but not lncRNA PTAR, may also act as sponges for miR-101. Thus, the newly identified AC004988.1-miR-101-FN1 axis may have a potential role in the iM subtype of OvCa, which warrants additional detailed studies.

## Conclusion

In summary, our systematic analysis uncovered a ceRNA regulatory network for the mesenchymal subtype of serous OvCa. We also highlighted the important role of lncRNA PTAR in promoting EMT in OvCa through the regulation of ZEB1 expression via miR-101. The findings from this study have significant implications regarding our understanding of OvCa metastasis. The effects of PTAR on the invasion-metastasis cascade suggest that it could be an effective target for anti-metastasis therapies in OvCa.

## Abbreviations

BH: Benjamini-Hochberg
CDH1: Cadherin-q, E-cadherin
ceRNA: competing endogenous RNA
DE: differentially expressed
EMT: Epithelial-mesenchymal transition
FDR: False discovery rate
FN1: Fibronectin 1
iE: integrated epithelial
IHC: Immunohistochemistry
iM: integrated mesenchymal
lncRNAs: long non-coding RNAs
OvCa: Ovarian cancer
PTAR: Pro-transition associated RNA
WT: Wild type
