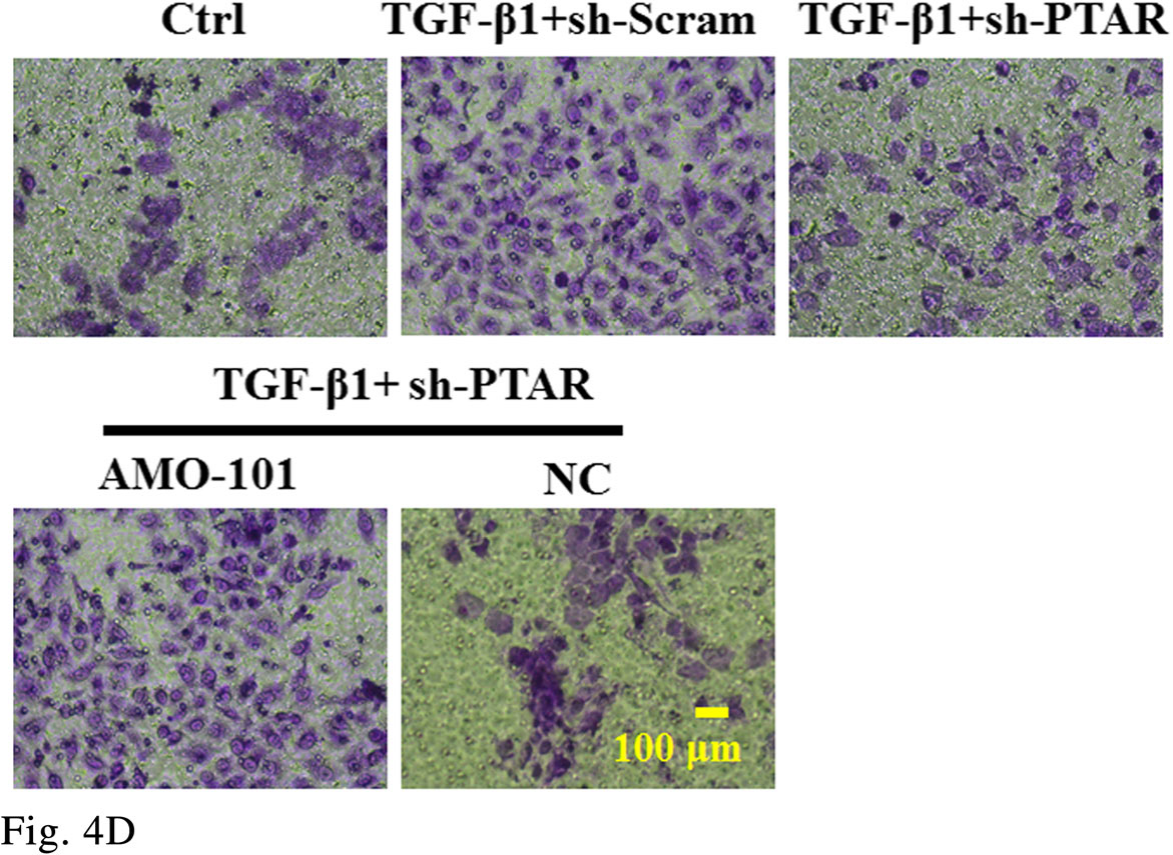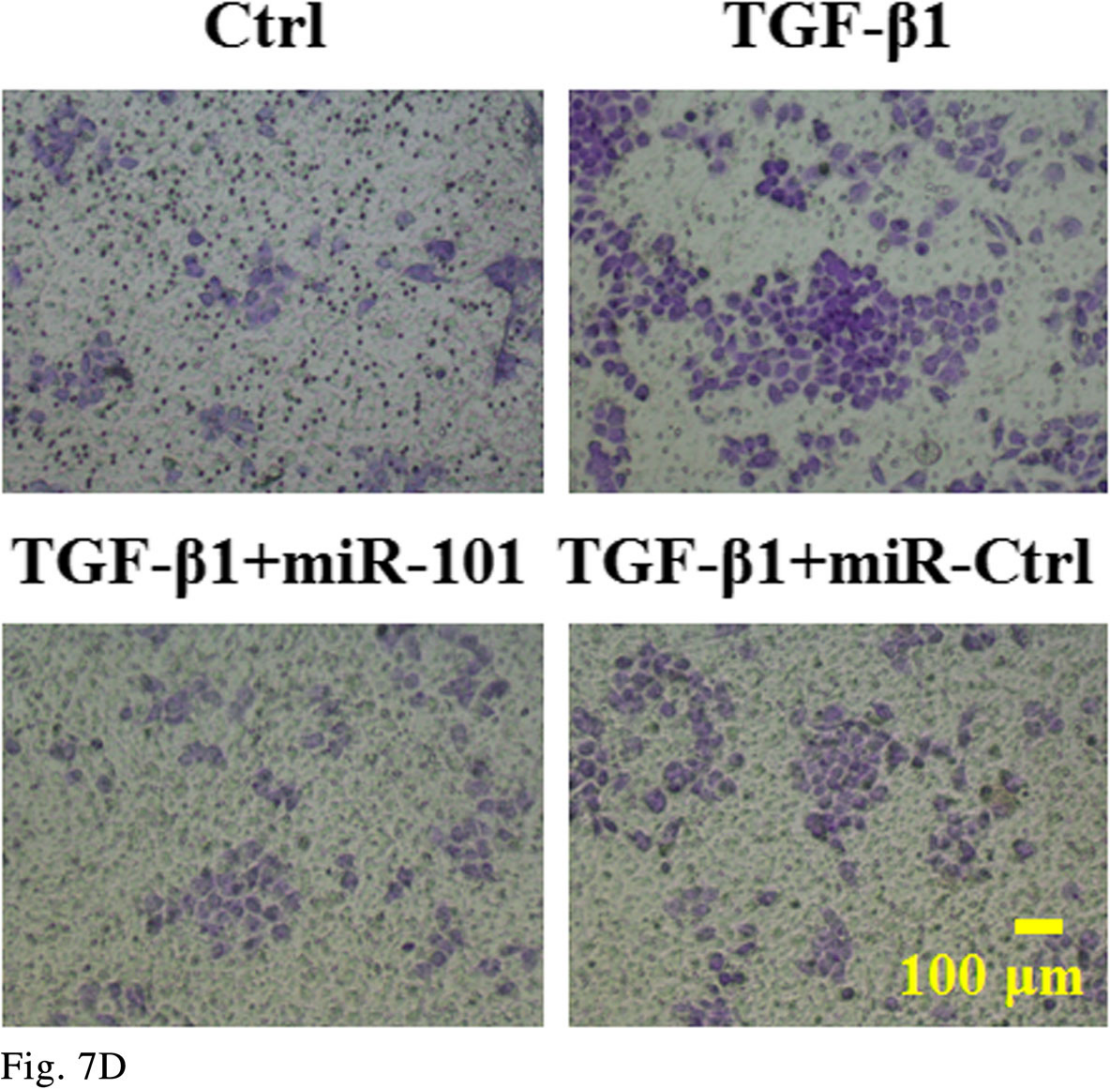# Correction to: LncRNA PTAR promotes EMT and invasionmetastasis in serous ovarian cancer by competitively binding miR-101-3p to regulate ZEB1 expression

**DOI:** 10.1186/s12943-021-01344-4

**Published:** 2021-04-09

**Authors:** Haihai Liang, Tong Yu, Yue Han, Hua Jiang, Chengyu Wang, Tianyi You, Xiaoguang Zhao, Huitong Shan, Rui Yang, Lida Yang, Hongli Shan, Yunyan Gu

**Affiliations:** 1 Department of Pharmacology (State-Province Key Laboratories of Biomedicine-Pharmaceutics of China, Key Laboratory of Cardiovascular Research, Ministry of Education), College of Pharmacy, Harbin Medical University, Harbin, China; 2 Translational Medicine Research and Cooperation Center of Northern China, Heilongjiang Academy of Medical Sciences, Harbin, 150081 China; 3 Department of Systems Biology, College of Bioinformatics Science and Technology, Harbin Medical University, Harbin, 150001 China; 4 Department of Systems Biology, College of Bioinformatics Science and Technology, Harbin Medical University, Harbin, 150086 China; 5 Training Center for Students Innovation and Entrepreneurship Education, Harbin Medical University, Harbin, 150086 China

**Correction to: Mol Cancer (2018) 17:119**

**https://doi.org/10.1186/s12943-018-0870-5**

Following publication of the original article [1], the authors identified some minor errors in image-typesetting in Figs. 3, 4 and 7. Specifically, the following panels have been corrected:

Figure 3B

Figure 4A

Figure 4D

Figure 7D

The corrected figures are given below. The corrections do not have any effect on the final conclusions of the paper.

**Figure 1:**
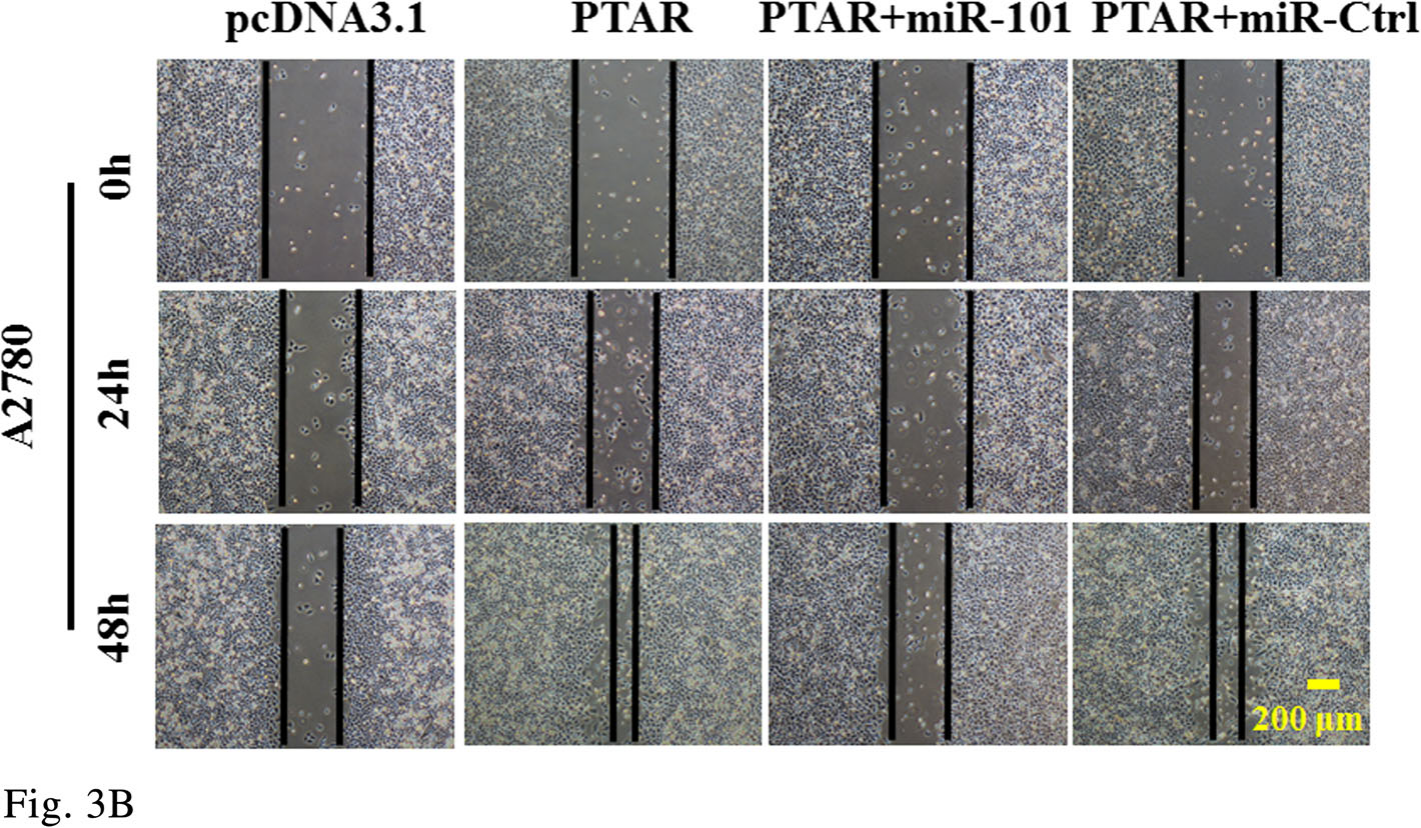

**Figure 2:**
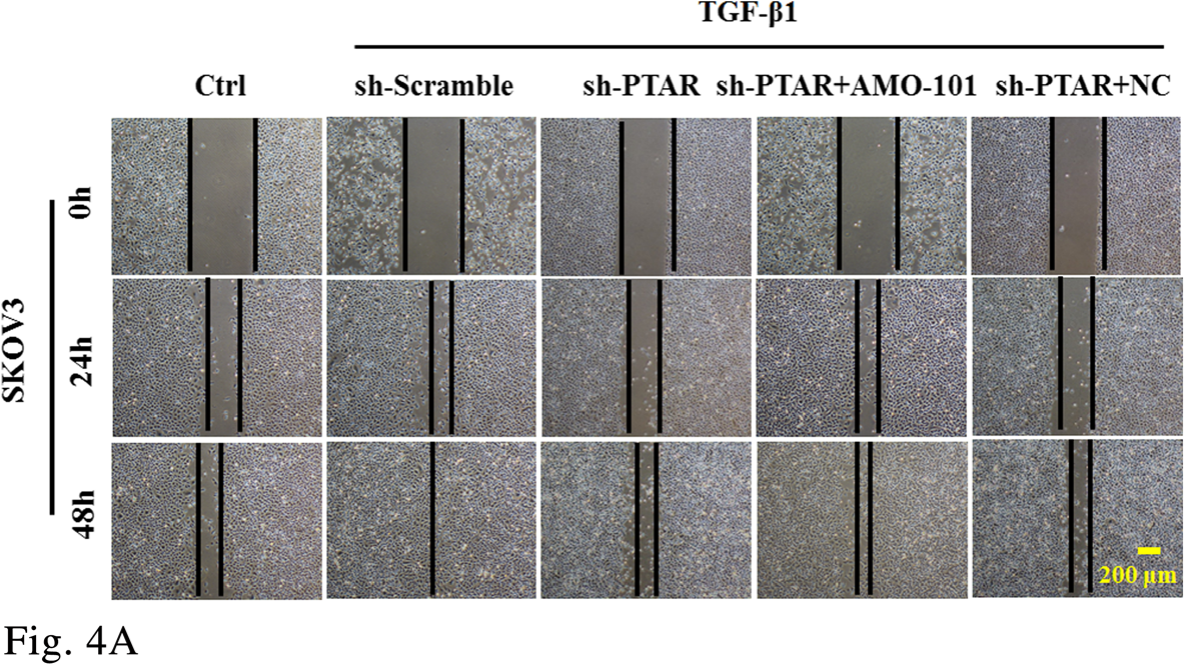

**Figure 3:**